# History of a Case of Tetanus, *in Which Large Quantities of the Tincture of Cantharides Were Ineffectually Employed*

**Published:** 1805-08-01

**Authors:** John Redman Coxe

**Affiliations:** Philadelphia


### History of a Case of Tetanus, *in which large Quantities of the Tincture of Cantharides were ineffectually employed; by* John Redman Coxe, M. D. *of Philadelphia.*

ELIJAH DUNN, aged fourteen years, was admitted on the 7th of June of the present year, into the Pennsylvania hospital, for an injury just received by a fall from a horse. A wound on the inner side of the right tibia, nearly two-thirds the length of the limb, and of considerable depth, together with one on the outer ankle of the left leg, but little more than skin deep, formed the extent of the accident. Both wounds were dressed with sutures and adhesive plasters, to favour union by the first intention. No fever or inflammation supervened; and the pain, considering the extent of the injury, was moderate. As he was bound in his bowels, on the 9th he took an ounce of Glauber's salts, which operated plentifully. The wounds were examined on the 13th, but no union was observed, and the discharge was profuse, but not purulent.

On the 14th, one drachm of bark with ten drops of elix. vitriol. were ordered to be taken three times a day; and the wounds were dressed morning and evening with poultices sprinkled with laudanum; by the 17th, the wounds assumed a more healthy appearance, granulations began to rise, but were soft and rather of a pallid hue, the discharge was thin and white, and a cicatrix had commenced. At this time he observed a difficulty of moving his lower jaw, but gave no information of it until the 18th, when the bark and vitriol were omitted, and one grain of opium was ordered three times a day, with a quart of white wine in the twenty-four hours. The disease seemed stationary till the 20th, at which time he opened his mouth with difficulty, and complained of stiffness of the muscles of the neck and abdomen,) which last were now nearly as unyielding as a board,) with slight spasms thro' the day. The wine appeared to exhilarate, and keep him free of that violent pain at the scrobiculus cordis, in general so severe in tetanic patients. The opium was now increased to a grain every two hours, and the wine ordered ad libitum. Spirit of turpentine was applied to the wounds, but excited no pain. It was then applied boiling hot, and even mixed with salt once or twice, with scarcely a sensation of pain. In the evening the symptoms had increased; thirty drops of tincture of cantharides were now given every hour, till he had taken four doses, when violent pains of the stomach and strangury, rendered its exhibition improper. The symptoms abated from the time this effect took place; but a tightness at the region of the stomach continuing, induced me to order a mustard poultice, with relief. Cantharides in powder were applied to the whole surface of the wounds, in hopes of exciting a more vigorous action in the languid vessels, but without effect. The pulse was increased in frequency, but diminished in force.

21st. The symptoms of strangury, &c. no sooner subsided, than the tetanic symptoms recurred, with permanent contraction of the muscles. The pulse beats 120, and weaker, and a degree of risus sardonicus exists. The jaws were close contracted,* and the head was drawn considerably

* Although his jaws were closed when awake, yet in sleep a complete relaxation of the muscles took place, the jaw falling upon the breast. But the tongue, which was perfectly still when awake, was, during sleep, in a constant state of spasmodic action, resembling the motion of the tongue of a snake. This was not however invariably the case.

siderably backwards. Ordered to apply flour of mustard to the sores, and take every two hours ten drops of tr. cantharidis in a glass of wine; continue the opium every two hours, and inject at the same interval of time, thirty drops of laudanum in two ounces of wine diluted with water up the rectum. If no effect is produced by the mustard, apply the butter of antimony to the sores.

22d. The butter of antimony was partially applied last night, and repeated this morning; the pain was considerable, but the action of the vessels was so torpid, that the slough was not separated for several days. The symptoms have abated: about three pints of wine have been taken by the mouth, and in the injections, and about ℥ij. of tr. of cantharides, producing a slight stranguary and pain of the intestines. He had one considerable convulsion last night, after which he slept a little.

23d. Last evening had a violent convulsion. His pulse is fuller, and his face is flushed, apparently from the wine taken; in swallowing his medicines, &c. he is obliged to be turned upon his belly, resting on his elbows, his head being drawn backwards considerably; and he is obliged to swallow slowly, to prevent a regurgitation of the fluid through the nostrils. He cannot now take the solid opium, I therefore ordered fifteen drops of laudanum every two hours in his wine; and as he has had no stool since the 19th, the wine injections were omitted for the present, and a common purging enema was administered. He passes his urine more frequently, but in smaller quantity and high coloured, with some scalding; it is, however, under his perfect control. The sores are tender, with a slight discharge. Continue the wine and laudanum, and increase the cantharides to thirty drops. If the strangury is not increased by the evening, apply a large blister to the region of the pubes. His food is chiefly panada, made rich with wine.

24th. The rigidity of the muscles is now universal, tho' he suffers but little pain, except when a violent convulsion seizes him, when his cries may be heard at a considerable distance. His mouth is opened with difficulty, and he bites his tongue frequently during sleep. Pulse 108; he slept tolerably; the glyster operated well yesterday, and the wine injections were resumed at five P. M. He does not retain them well; I therefore ordered them in diminished doses; viz. ℥ß of wine and as much water, with forty drops of laudanum. About a quart of wine has been used since yesterday. The blister was applied as directed, and rose rose well, with considerable pain, but no increase of strangury. Ordered fifty drops of tr. of cantharides, and thirty of laudanum every two hours.

25th. Butter of antimony was applied this morning over the whole extent of the sores, producing great pain of short duration; skin is generally moist, and he appears slightly better, opening his mouth wider.

26th. Half past nine. The symptoms were aggravated; about five last evening a violent convulsion seized him. He does not take as much wine as is desirable. Slight spasms attack him every five minutes; a miliary eruption has appeared, chiefly about the neck and body; pulse varied much during the day, sometimes full and slow, at others, weak and frequent. Pledgets of linen constantly wet with brandy were applied to the sores. A glass of wine was ordered every hour, and the tinctures of cantharides and opium as before. If no effect occurs by noon, give fifty drops of laudanum every hour with the wine, and fifty drops of tinctuie of cantharides. Increase the laudanum in the glysters to fifty drops.

By noon the increased doses were commenced, and persevered in, till he had taken about four hundred drops of laudanum and as much cantharides, and half the quantity by injection. At six P. M. a violent convulsion attacked him, lasting half an hour, and he began to grow comatose, with now, (eight P. M.) a degree of stertorous breathing, and that extreme irritability produced by laudanum, awaking if touched, in convulsions, and affected with frequent tremors. No relaxation of the jaws; the pulse is good. Ordered to omit all his medicines, to apply a large blister over his head; and if the symptoms of coma, &c. increase, give ʒß of white vitriol and as much ipecacuanha in divided doses, to excite vomiting. Should the convulsions increase by ten o'clock, dash over him two buckets full of cold water, and repeat it if serviceable, every two or three hours.

27th. ten A. M. Blister drew well. The attendants kept him constantly awake, and in a few hours the effects of the laudanum subsided, though he yet continues very drowsy. The jaws are still close, but on the whole he seems better. He makes water often, but in small quantity, with some scalding, and high coloured, which is all the tendency to strangury that has occurred since the first doses of cantharides. The tr. of cantharides was again resumed in doses of *one hundred* drops every hour, until by six P. M. he had taken *one thousand* drops, *without the*

*slightest*

*slightest effect apparent.* It was therefore discontinued altogether. Wine was ordered ad libitum in the evening, and fifteen drops of oil of amber to be given every hour, augmenting each dose; he took but one dose, as he said it nearly strangled him, and would not try a second.

28th. This morning he began early the use of volatile alkali in doses of five grains every hour; three or four doses have been taken without any visible effect. He had one violent convulsion last night and two this morning, at other times remaining free of pain though quite rigid, and having frequent twitches of the muscles. The sores are more inflamed around the edges, with considerable pain when touched, the sensibility appearing to be perfectly morbid. During the night the urine came away involuntarily. He has now, however, the complete command of it. I now determined to try cold water by affusion, in which I was sanctioned by Dr. Rush, who came in at this period; his pulse was 104, and tolerably vigorous. At noon a bucket full of cold water was poured through a cullender over him as he lay in bed, on an oil cloth, and he was suffered to remain in it for two minutes before he was wiped; he said it felt cool and comfortable; the rigidity of the muscles appeared to abate, and a glow of heat succeeded. The eruption, noticed on the 26th, had now extended itself to the thighs. I ordered the cold water to be continued every two hours, increasing the coldness and quantity if it proved useful. These favourable symptoms continued for about half an hour, but the disease appeared to return with redoubled violence, especially on attempting to drink, which nearly strangled him, and prevented the use of any thing but a little wine. The use of the water was suspended. At eight P. M. the pulse flagged considerably; I ordered the wine to be continued ad libitum, with thirty drops of laudanum every two hours; to rub in ʒß of strong mercurial ointment every hour into the thighs, and five grains of calomel every two hours into the gums.

29th. The laudanum last night gave him almost immediate relief, and he dozed, without spasms during the night, being awakened regularly to take his medicines. Butter of antimony was applied last evening, but only partially, as it gave him exquisite pain, and appeared to augment the spasms. The sores have a sloughy appearance; continue to wet them with the spirits. The eruption has increased since yesterday, some being of the size of a small pin's head and filled with a whitish matter. Urine is passed without much difficulty or scalding, in larger quantity, and less coloured. A pulsation of the heart, which was first observed three or four days ago, has become very violent, and is visible at a considerable distance from him. It was remarked to be always greatly increased during his convulsions, but it is now more uniform. The pulse both here and at the wrist beats about 112, but is strongest in proportion at the heart. He swallows with more ease this morning, and the muscles of the back appear less rigid, as the arch formed when he is turned on his face to drink, is less than heretofore, and he now can drink slowly when lying on his back. His gums seem slightly affected by the mercury, of which he has taken about ℥ß, and nearly half a pound of the ointment has been faithfully rubbed in. As he had had no stool since the 23d, I ordered a common glyster, which operated once very copiously, with apparent benefit, the spasms recurring less frequently, and he can open his mouth nearly one half inch. The muscles of the body generally, are more relaxed, so as to give great hopes of a favourable issue. I prescribed thirty drops of laudanum every hour, to continue the ointment every two hours, and desired the calomel to be omitted.

At two P. M. there was a remission of all the symptoms, and he seemed apparently better than he had been since his first attack; his spirits were high, and he eat voraciously. Appearances continued thus favourable till about six P. M. when, in the act of drinking, a most violent convulsion seized him, in which his body was bent nearly into the form of a semi-circle backwards; the muscles of the neck were particularly affected. This convulsion lasted without any abatement for about an hour, his face becoming very livid and respiration very torpid; the pulse at the wrist being scarcely perceptible, whilst the convulsive motion of the heart was greatly augmented; and at seven o'clock death released this unfortunate victim from his accumulated sufferings.

### Dissection.

At ten A. M. of the 30th, fifteen hours after his decease, the body was opened, the muscles remaining still rigid, though several purple blotches over the body and extremities, appeared to denote the tendency to putrefaction. The abdomen was much distended from flatus in the alimentary canal. The adipose matter was very small, the fœtor considerable. Of the omentum, scarcely any thing remained mained but a thin transparent membrane. The stomach externally was sound, but internally were several small appearances of inflammation, especially near the pylorus. It contained the panada he had last taken, mixed with mucus, in about three-fourths of a pint. The spleen was altered slightly in its colour, being of a deeper leaden hue than natural. The liver was sound; the gall-bladder large and distended with yellow bile, which had tinged the adjoining parts very considerably. The kidneys and ureters were sound; and the bladder, containing about two ounces of urine, was contracted, and its coats thickened, especially at its fundus. No inflammation appeared in it. The thoracic viscera were sound, except the heart, which appeared to be smaller than usual, and to be still under the influence of that spasmodic action which existed so powerfully in his last moments. The carnæ columnæ especially appeared to be in a state of strong contraction, being permanently rigid, with none of that flaccidity, which might have been expected so long after death had taken place. The blood was not in coagula, but dissolved like molasses, as in animals killed by lightening, appearing to indicate, that the whole muscular fibres of the arterial system had partaken of the general spasmodic action.

On examining the throat, &c. the œsophagus was perfectly sound, but the *epiglottis and trachea were highly inflamed, especially the last, increasing in redness as we approached to the lungs.*

In the course of the disease, the patient took about 2400 drops of tincture of cantharides, about 2000 of tincture of opium, besides the quantity used in the glysters, and nearly three gallons of wine.

---

I do not recollect any case recorded of dissection after death from tetanus. It remains therefore to be ascertained by future observation, whether inflammation of the trachea always exists. From the neck being a part generally first affected in this disease, we might à priori judge this to be the case. One circumstance to be remarked however is, that in the above case no difficulty occurred in swallowing fluids, (except that only a small portion at a time could be taken) which might not have been expected, considering the highly inflamed state of the adjoining parts.

As all the muscles partake of the spasmodic action of this disease, even the bladder, intestines and heart, may we not reasonably conclude, that the arteries partake in common of the same state, in a greater or less degree?

I 4 Will

Will not this state of the arteries, account for the apparently weak and quick pulse, which is common in tetanus, and which is seldom excited to febrile action and fulness, even by the largest doses of wine and laudanum? And may we not hence also explain the great tendency to solution of the blood, which is noticed in this disease? What would be the effect of bleeding in small quantities, and gradually increasing the quantity drawn, in removing this spasmodic state? Would not a vigorous action of the vessels be thereby excited, and an inflammatory crust produced on the blood; as has been observed in some very malignant cases of fever, where the depressed pulse and dissolved blood have gradually given way to violent action and sizy blood, requiring a continuance of the use of the lancet with greater freedom, to subdue this more active, though less dangerous state of fever.

Is it not probable that the strangury (except in the very first instance) was owing to the disease, and not to the medicine taken, as it was always very trifling, even under the large and repeated doses of the tincture of cantharides? And has not this medicine rather proved beneficial by the inflammation it excites in the stomach and bowels, than by its action on the urinary organs? for otherwise we must suppose the strangury from the medicine and from the disease to differ greatly in their effects upon the system.

*August* 1, 1804.

